# Phase 1 results of a phase 1b/2, multicenter, open-label trial to evaluate safety and efficacy of talimogene laherparepvec (T-VEC) and ipilimumab (ipi) vs ipi alone in previously untreated, unresected stage IIIB-IV melanoma

**DOI:** 10.1186/2051-1426-1-S1-P84

**Published:** 2013-11-07

**Authors:** Igor Puzanov, Mohammed Milhem, Robert Andtbacka, David Minor, Omid Hamid, Ai Li, Ari VanderWalde, Howard Kaufman

**Affiliations:** 1 Vanderbilt University, Nashville, TN, USA; 2 University of Iowa, Iowa City, IA, USA; 3 Huntsman Cancer Institute, Salt Lake City, UT, USA; 4 California Pacific Center, San Francisco, CA, USA; 5 The Angeles Clinic and Research Institute, Los Angeles, CA, USA; 6 Amgen Inc., Thousand Oaks, CA, USA; 7 Rush University, Chicago, IL, USA

## Introduction

T-VEC is a herpes simplex virus type-1-derived oncolytic immunotherapy designed to produce GM-CSF and to selectively replicate in and lyse tumors to induce a systemic anti-tumor immune response. A phase 3 study of T-VEC in Stage IIIB-IV melanoma demonstrated a significant improvement in ≥ 6 mo durable response rate vs GM-CSF and a tolerable safety profile.

## Methods

In this 2-part study (NCT01740297), phase 1b evaluated the safety of T-VEC in combination with ipi as assessed by the incidence of dose-limiting toxicities (DLTs) at full doses of both agents. For 6 to 9 patients (pts) evaluable for DLT, approximately 18 pts were to be enrolled in phase 1b. DLTs were defined as any grade (gr) ≥ 3 immune-related adverse event (AE) or gr ≥ 4 AE of any etiology occurring between the first dose of ipi and 6 wks after. The incidence of DLTs was required to be ≤ 1 of the first 6 evaluable pts or ≤ 2 of the first 9 evaluable pts (if 2 DLTs were seen in the first 6 pts). Key entry criteria were previously untreated, unresectable Stage IIIB-IV melanoma, ECOG 0-1, measurable disease, and ≥ 1 injectable cutaneous, subcutaneous, or nodal tumor. T-VEC was administered by intralesional injection at ≤ 4 mL of 10^6^ plaque forming units (PFU)/mL for the first dose, then 10^8^ PFU/mL on day 1 of wk 4 and thereafter. Ipi 3 mg/kg q3w was administered as 4 infusions starting at wk 6. Pts received T-VEC until development of DLT, all injectable tumors have disappeared, disease progression per the Immune Related Response Criteria, or treatment intolerance. Phase 2 will evaluate the safety and efficacy of ipi vs T-VEC+ipi in a randomized fashion.

## Results

To date, 19 pts have enrolled (13 pts received ≥ 1 dose of T-VEC); 9 pts are evaluable for DLT. All DLT evaluable pts received at least 4 doses of T-VEC and 2 doses of ipi by the time of DLT cutoff (6 wks post first ipi dose). No DLTs were observed in evaluable pts. Serious AEs were reported in 1 of 19 pts to date (gr 3 nausea and abdominal distention in week 11 of treatment). Two partial responses were reported by wk 12 in the 9 DLT evaluable pts. Data will be updated at the meeting.

## Conclusions

Advancement into the randomized phase 2 is planned pending Data Review Team recommendations.

